# Assessment of Bone Health in Adult Patients with Inflammatory Bowel Disease: A Single-Center Cohort Study

**DOI:** 10.3390/jcm14113933

**Published:** 2025-06-03

**Authors:** María Cortés-Berdonces, Beatriz Arberas, Marina de la Fuente, Israel J. Thuissard, Fernando Marín

**Affiliations:** 1 Department of Endocrinology, Hospital Universitario Ruber Juan Bravo, 28006 Madrid, Spain; 2 Department of Medicine, Faculty of Biomedical and Health Sciences, Universidad Europea de Madrid, 28670 Madrid, Spain; 3 Department of Gastroenterology, Hospital Universitario Ruber Juan Bravo, 28006 Madrid, Spain; 4 Department of Radiology, Hospital Universitario Ruber Juan Bravo, 28006 Madrid, Spain; 5 Department of Endocrinology, Hospital Universitario Quirónsalud, 28006 Madrid, Spain

**Keywords:** trabecular bone score, bone ultrasound, 3D-DXA, inflammatory bowel disease, Crohn’s disease, ulcerative colitis

## Abstract

**Background:** Most of the studies that have investigated bone quality in inflammatory bowel disease (IBD) have utilized dual-energy X-ray absorptiometry (DXA). We assessed the bone status of IBD adult patients using a comprehensive array of non-invasive techniques. **Methods:** Fifty IBD patients (30 women) and 50 healthy volunteers—matched for age, gender, and body mass index—were prospectively recruited. Areal bone mineral density (aBMD) at the anteroposterior and lateral spine and the proximal femur was measured by DXA, including vertebral fracture assessment (VFA). Trabecular bone score (TBS), calcaneal quantitative ultrasound (QUS), volumetric bone mineral density (vBMD), and cortical thickness were assessed in the proximal femur with 3D-DXA. A comprehensive laboratory panel of calcium metabolism and bone turnover markers was included. **Results:** Twenty-nine and 21 patients were diagnosed with ulcerative colitis (UC) and Crohn’s disease (CD), respectively. VFA identified vertebral fractures in two IBD patients and no controls. No statistically significant differences were observed in TBS, aBMD, and vBMD between IBD and healthy controls. After excluding one predefined outlier, broadband ultrasound attenuation (BUA) showed lower values in IBD vs. controls [103.6 ± 14.3 vs. 111.3 ± 19.5 (*p* = 0.033)]. QUS analysis revealed statistically lower values in the CD group compared to controls. We found a positive correlation between all the QUS parameters with aBMD and vBMD. **Conclusions:** In our study of IBD subjects, most of whom had mild or quiescent disease, we did not observe significant bone quality deterioration. QUS was the only technique that showed lower values in IBD patients, especially in CD.

## 1. Introduction

Inflammatory bowel disease (IBD) encompasses two main clinical forms: Crohn’s disease (CD) and ulcerative colitis (UC). Both conditions involve immune-mediated inflammation of the gastrointestinal tract and are associated with a substantial number of digestive and systemic comorbidities [1]. The diagnosis of IBD is considered a risk factor for bone loss and an increased risk of fractures, particularly vertebral fractures, has been reported in these patients [2,3]. The estimated prevalence of osteoporosis in individuals with IBD falls within the range of 2–15% [4], with an incidence of osteoporosis that is twice as high as that in the control population [5]. The severity and extent of inflammation are two factors that influence bone metabolism in IBD, impacting the elevation of proinflammatory cytokines such as tumor necrosis factor alpha (TNF-α) and interleukins (IL) IL-1β, IL-6, and IL-17, which exert a detrimental effect on bone tissue [5,6,7]. Other risk factors for bone loss in IBD are the use of glucocorticoid therapy, malnutrition, short bowel syndrome resulting from intestinal resections, low calcium intake, vitamin D deficiency, and diminished muscle mass and low peak bone mass in children and young adults with the disease [5,6,7,8]. Some studies have noted that CD presents a higher prevalence of osteoporosis and lower bone mineral density (BMD) than UC [4,9,10]. This difference appears to be attributed to the specific small bowel disease in CD, as the involvement of the small bowel or its resection is a significant risk factor for bone loss [7]. In fact, several studies [7,9,11] and a recent meta-analysis [4] that analyzed results separately for CD and UC have shown no significant effects of UC on an increased risk of osteoporosis nor a higher risk of fractures.

Most studies that have assessed bone quality in IBD patients have used areal dual-energy X-ray absorptiometry (DXA) as the primary tool for measuring bone mass. However, although BMD measured with DXA is considered the gold standard for diagnosing osteoporosis, it only partially explains bone quality properties and the fracture risk. In recent years, DXA scanning has seen advancements, including vertebral fracture assessment (VFA) using lateral views of the thoracolumbar spine [12]; hip structural analysis, which utilizes hip cross sections to ascertain bone strength [13]; and 3D-DXA for evaluating femoral shape, cortical thickness, density distribution and trabecular macrostructure [14], and trabecular bone score (TBS), which provides a measure of bone quality and serves as a surrogate of bone microarchitecture [15]. Additionally, quantitative bone ultrasound (QUS) is a radiation-free method that allows for the analysis of some physical properties of bone tissue that, in turn, are important determinants of bone stiffness, load failure, and fracture risk by providing additional information to bone mass alone [16]. Both TBS and QUS predict osteoporotic fractures independently of areal BMD [17,18]. These new technologies have rarely been employed to evaluate IBD patients, and studies using TBS as the primary tool have provided some conflicting results [19,20]. As a result, our study primarily aimed to investigate the bone quality properties of IBD patients compared to a matched group of healthy subjects, with TBS as the primary technology. Additionally, we assessed areal BMD (anteroposterior and lateral spine and femoral), VFA, hip 3D-DXA, QUS, and biochemical markers of bone turnover. Secondarily, we analyzed the differences between UC and CD patients and the correlations between these bone parameters.

## 2. Materials and Methods

### 2.1. Study Population

Fifty adult patients with IBD and 50 healthy volunteers without IBD or known metabolic bone disease, adjusted for age, sex, and body mass index (BMI), were prospectively recruited between October 2021 and January 2023 at the tertiary care outpatient clinics of the Departments of Gastroenterology, Rheumatology and Endocrinology of the Hospital Ruber Juan Bravo, Madrid, Spain. The diagnosis of IBD and its specific subtype was previously histologically verified and accessible in the patients’ medical histories. Subjects in the control group were identified and recruited in the same departments as IBD patients. Exclusion criteria included individuals with other causes of metabolic bone disease (e.g., Paget’s disease, severe chronic renal failure, metastatic bone disease, multiple myeloma, and hypercalcemia) and those who had been immobilized for more than three months in the preceding year. Pregnant individuals, as well as those with anatomical foot alterations that could interfere with calcaneal ultrasound, individuals taking treatments affecting bone metabolism, and those participating in clinical trials involving drugs for osteoporosis or IBD at the time of recruitment were also excluded. Additionally, individuals with chronic diseases affecting bone metabolism or treated with drugs that negatively impact bone quality, such as glucocorticoids, anticoagulants, antiretrovirals, anti-oestrogens, or antiandrogens, were excluded from the non-IBD control group. The study was conducted in accordance with the ethical principles outlined in the Declaration of Helsinki. This study was approved by the Ethics Committee of Fundación Jiménez Díaz in Madrid, Spain. All participants provided informed consent prior to their inclusion in the study.

### 2.2. Clinical Evaluation and Bone Assessment Techniques

Baseline study visits involved collection of demographics, and clinical information, including age, gender, anthropometric measurements, risk factors for osteoporosis, history of previous fractures due to low impact trauma, estimated calcium intake, and the use of calcium and/or vitamin D supplements.

The 10-year absolute risk of hip fracture (HF) and major osteoporotic fractures (MOF) was calculated by FRAX for both groups in subjects older than 40 years, adding the BMD femoral neck values and with and without TBS results. Osteoporosis was defined as a value for areal DXA BMD T-Score ≤ −2.5 SD at the femoral neck, total hip, or spine for subjects aged 50 and older. In subjects less than 50 years of age, a Z-score ≤ −2.0 SD was applied. Within the IBD patient cohort, disease data were collected such as time of evolution, disease activity, history of previous bowel resection, current and past therapies for IBD, and the use of systemic and topical corticosteroids. We employed the Montreal classification for Inflammatory Bowel Disease (Supplementary Tables S1 and S2) [1].

BMD [expressed in grams per square centimetre (g/cm^2^) was assessed by DXA at the proximal femur and the lumbar spine (L1–L4), taking anteroposterior and lateral measurements with a Prodigy Advance Full Encore version 11.x equipment (General Electric, Boston, MA, USA). T-scores were calculated for subjects aged 50 years and above, while Z-scores were computed for those subjects younger than 50 years old. The precision estimates [coefficient of variation (CV)] at our center are CV 1% for the lumbar spine and total hip. A quantitative morphometric analysis of the spine (T4–L4) was performed using the “VFA Dual Vertebral Assessment H8650DA/DM” software (General Electric, Boston, MA, USA).

The microarchitectural parameters of the lumbar spine were evaluated with TBS (INsight v3.0) (Medimaps Group, Geneva, Switzerland). Volumetric bone mineral density (vBMD) [expressed in grams per cubed centimetre (g/cm^3^)] and cortical thickness were assessed in the proximal femur with 3D-DXA (3D-SHAPER research v2.11.2 software) (Galgo Medical, Barcelona, Spain). Detailed information about the modeling method used in the software has been published elsewhere [14]. The 3D-DXA CV is similar to DXA, and least significant changes (LSC) are 10,39 mg/cm^3^ for integral vBMD and 9.64 mg/cm^3^ for trabecular vBMD [21]. For quantitative calcaneal bone ultrasound parameters, broadband ultrasound attenuation (BUA) and speed of sound (SOS) were evaluated with a SONOST 3000 machine (OsteoSys Co, Seoul, South Korea) in the left calcaneus, and the results were combined to provide a single parameter called bone quality index (BQI) using the following equation: BQI = 0.72 × SOS + 0.41 × BUA − 1045. Daily quality control was performed with acoustic phantoms provided by the manufacturers. The reproducibility for BUA and SOS is ≤1% CV (manufacturer’s data).

Parameters of calcium-phosphate metabolism were analyzed in fasting state and included total calcium, albumin, phosphorus, magnesium, 25-OH-vitamin D (Cobas, Roche Diagnostic, Manheim, Germany), intact parathyroid hormone (iPTH) (Cobas, Roche Diagnostic, Manheim, Germany), creatinine, estimated glomerular filtrate (CKD-EPI), ß-isomer of carboxy-terminal telopeptide of type I collagen (ß-CTx, Crosslaps) (Cobas, Roche Diagnostic, Mannheim, Germany), N-terminal propeptide of type I procollagen (P1NP) (Cobas, Roche Diagnostic, Mannheim, Germany), and urine calcium/creatinine ratio. Measurements of P1NP and beta-CTx were made using the Elecsys electrochemiluminescence immunoassay System (Roche, Indianapolis, IN, USA).

### 2.3. Statistical Analysis

Assuming a 50-point difference in TBS values between the two study groups and assuming an 80% statistical power (β = 0.2) and a standard deviation (SD) of 100, we calculated a total of 50 subjects in each group. This estimation was deemed clinically significant, considering the results from several cohort studies that analyzed TBS in several other disorders [22,23,24,25,26,27,28,29,30,31,32,33], since conclusive TBS data for adults with IBD were lacking. Differences were considered statistically significant at *p* values less than 0.05 with bilateral contrast. Categorical variables were presented as numbers (n) and percentages (%). Quantitative variables were presented as mean ± standard deviation (SD) or as median and interquartile range (IQR), depending on the normal distribution of the data. For the comparison of quantitative variables, Student’s *t*-tests for independent samples were used when the data followed a normal distribution, or Mann–Whitney U test if the data did not conform to normality. Likewise, for comparing categorical variables, Chi-square tests or Fisher’s exact tests were used. No correction for multiple testing was performed. Correlations of continuous and categorical variables were analyzed with Pearson’s test for normal data and Spearman’s test for data that did not follow a normal distribution. Subsequently, the IBD group was subdivided into two categories, UC and CD, and the differences with respect to controls were analyzed by analysis of variance (ANOVA). In instances where outlier values, defined as those exceeding or falling below 3.0 standard deviations for each quantitative variable, were encountered, we reported the results both with and without the inclusion of these outliers, only if their exclusion altered the conclusions. Otherwise, the results were reported with the inclusion of outliers. Statistical analysis was performed with SPSS version 23.0 (IBM Corp., Armonk, NY, USA).

## 3. Results

### 3.1. Characteristics of the Study Populations

**Figure 1:**
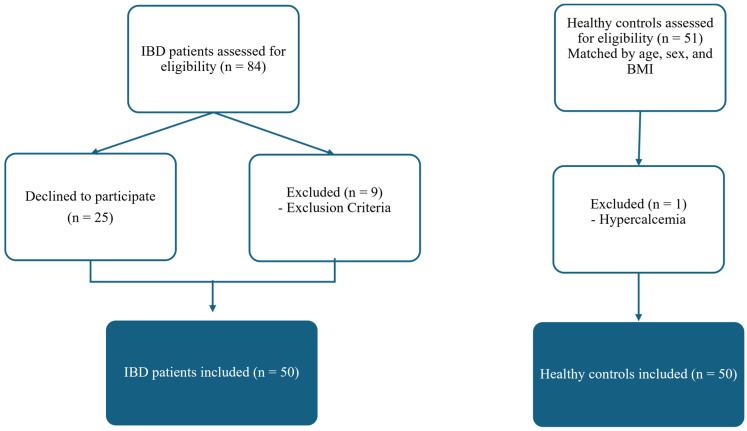
Flowchart of patient recruitment and selection, including exclusion criteria and final group assignment.

Eighty-four consecutive patients with IBD were screened. Nine had protocol defined exclusion criteria, and 25 refused to participate. The patient selection process is summarized in Figure 1. The IBD group included 29 patients with UC and 21 patients with CD. An age, sex, and BMI- matched healthy control was prospectively enrolled for each IBD patient. The demographic characteristics, risk factors, and laboratory findings of the study subjects are presented in Table 1. There were 20 (40%) males and 30 (60%) females in each group. IBD patients and controls were comparable in age (mean + SD): 52.0 ± 13.3 vs. 52.0 ± 4.3, and BMI: 25.4 ± 4.1 vs. 26.1 ± 4.3 kg/m^2^, respectively. The two groups did not differ in terms of smoking, alcohol consumption, menopausal status, previous history of low-trauma fracture, hip fracture history in parents, use of calcium and vitamin D supplements, or calcium intake (Table 1). The biochemical markers of bone metabolism, including bone turnover markers, did not show clinically relevant differences between groups. Statistically significant differences were observed in albumin and albumin-corrected calcium levels, which were slightly lower in healthy controls and patients with IBD, respectively, but without clinical significance (Table 1).

The risk of hip fracture and major osteoporotic fracture was calculated by FRAX for both groups (Table 1) with no statistically significant differences, even when the calculation was adjusted for TBS. A higher calculated risk of hip and mayor osteoporotic fractures was observed in patients with CD compared with the UC subgroup.

Table 2 shows the disease specific characteristics of IBD patients. The mean duration of the disease was 10.6 ± 11.7 years. According to the Montreal classification for IBD, 21 (72%) UC patients were in clinical remission (S0), 3 cases (10%) showed mild UC (S1), 4 cases (14%) showed moderate UC (S2), and only 1 case (3%) presented severe UC (S3). Nine patients (31%) presented with pancolitis (E3) in the UC group. In patients with CD, 15 cases (71%) presented non-stricturing, non-penetrating behavior (B1), 2 cases (10%) presented stricturing behavior (B2), and 4 cases (19%) showed penetrating (B3) behavior. Six (29%) patients in the CD group had ileocolonic involvement (L3). Four (8%) patients had received previous surgery, all of them in the CD group. The drug therapies for IBD are detailed in Table 2. Six (12%) patients had received corticosteroid treatment in the 3 months prior to the study. Overall, 54% of the IBD patients had received systemic corticosteroid treatment at any time during the disease, with a cumulative dose (mean ± SD) of 3.28 ± 2.09 g, with no differences between the UC and the CD groups.

**Figure 2:**
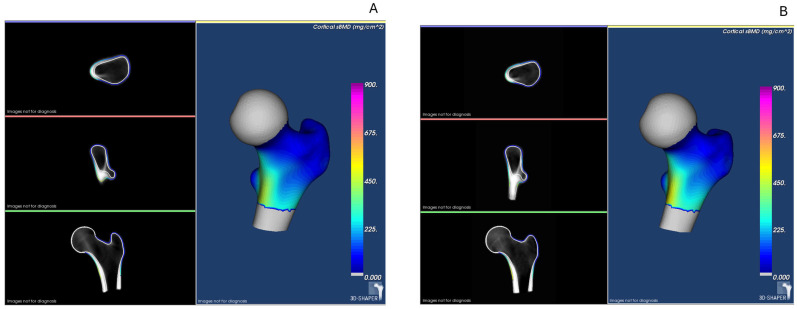
Representative 3D-DXA images illustrating bone structure and geometry assessment. (**A**) IBD patient: cortical vBMD 765.21 mg/cm^3^, trabecular vBMD 160.50 mg/cm^3^, integral vBMD 299.92 mg/cm^3^, and cortical thickness 1.760 mm. (**B**) Control subject: cortical vBMD 835.51 mg/cm^3^, trabecular vBMD 172.65 mg/cm^3^, integral vBMD 316.88 mg/cm^3^, and cortical thickness 1.751 mm. *Note: Values correspond to individual cases and are shown for illustrative purposes only*.

In the first part of the analysis, we compared patients with IBD with healthy controls. Overall, although all the numerical values of the bone quality parameters were lower in the IBD group, no statistically significant differences were found in the results of TBS, areal BMD at the lumbar spine and proximal femur, volumetric BMD, and cortical thickness at the hip, nor in the quantitative ultrasound parameters between the two groups (Table 3). The difference in spine aBMD between the IBD and control groups, evaluated using lateral DXA, was greater than with the antero-posterior view (−7.2% vs. −1.6%, respectively). The predefined exclusion of one outlier in the BUA values resulted in a statistically lower value in the IBD group (Table 3). More patients in the IBD group were diagnosed with osteoporosis according to the aBMD T- or Z-score cut-off values compared with the control group (20% vs. 6%, *p* = 0.037). A visual representation of the 3D-DXA analysis is provided in Figure 2, showing representative cases from each study group.

**Figure 3:**
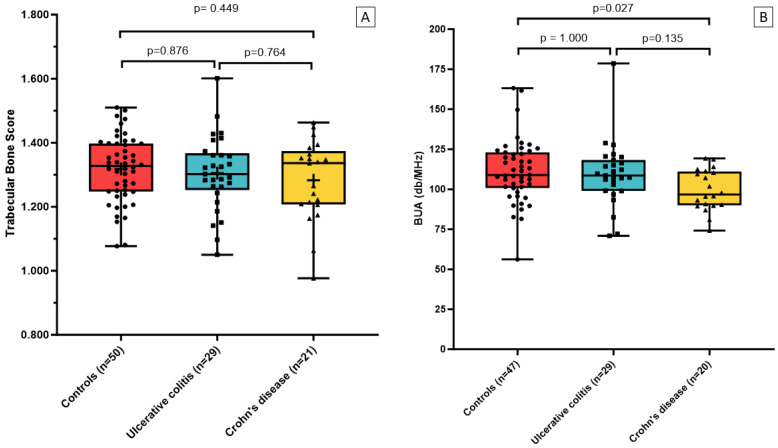
Comparison among controls, ulcerative colitis, and Crohn’s disease. (**A**) trabecular bone score. (**B**) BUA (db/MHz). Red boxes represent control subjects, blue boxes represent patients with ulcerative colitis, and yellow boxes represent patients with Crohn’s disease. Each dot, square, or triangle corresponds to an individual participant. Bold horizontal lines indicate the median and box edges show the interquartile range. Abreviations: TBS (trabecular bone score), BUA (broadband ultrasound attenuation).

The comparison of the bone assessments of UC and CD patients revealed no statistically significant differences in TBS nor areal and volumetric BMD (Figure 3). However, the analysis of calcaneal ultrasound variables showed statistically lower values of BUA and BQI in the CD group compared with the healthy controls and UC patients (Figure 3). VFA analysis detected three vertebral fractures in two patients in the IBD group and none in the control group.

### 3.2. Correlations Between Bone Assessment Techniques

**Figure 4:**
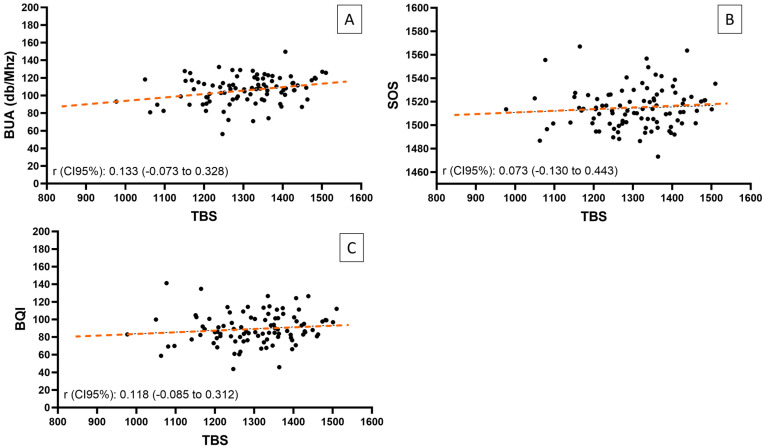
Pearson correlation between TBS and QUS parameters. (**A**) Correlation between TBS and BUA (outlier excluded). (**B**) Correlations between TBS and SOS. (**C**) Correlation between TBS and BQI. Each point represents an individual participant. Orange dashed lines indicate linear regression fits. BUA (broadband ultrasound attenuation), SOS (speed of sound), and BQI (bone quality index).

**Figure 5:**
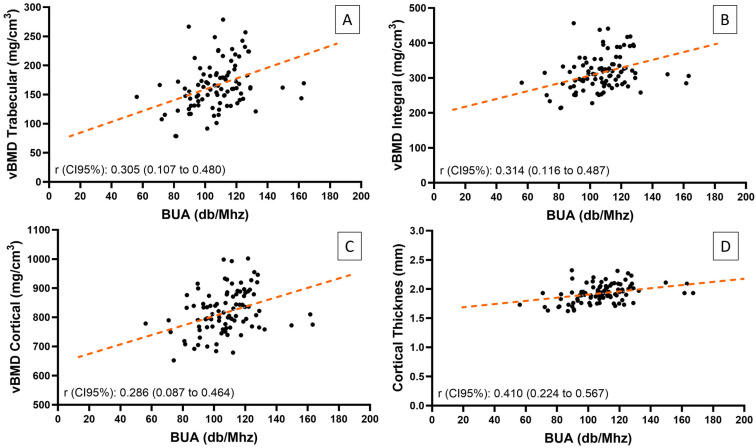
Pearson correlation between BUA and 3D-DXA. (**A**) Correlation between BUA and vBMD trabecular. (**B**) Correlation between BUA and vBMD integral. (**C**) Correlation between BUA and vBMD cortical. (**D**) Correlation between BUA and cortical thickness. Each point represents an individual participant. Orange dashed lines indicate linear regression fits. BUA (broadband ultrasound attenuation) and vBMD (volumetric bone mineral density).

Table 4 shows the correlations between different bone assessment techniques in the total population analyzed. After excluding one outlier, the weak correlation observed between TBS and BUA in the full cohort was not confirmed (r = 0.133, *p* = 0.205) (Figure 4, Table 4). No significant correlation was found between TBS and areal or volumetric BMD or QUS parameters. Conversely, all three parameters of calcaneal QUS were positively correlated with all proximal femur 3D-DXA parameters, as well as with areal BMD (anterior-posterior and lateral). Regarding the correlations between BUA and 3D-DXA variables, we found significant correlations when the BUA outlier was removed from the analysis (Figure 5), the strongest correlation being between BUA and cortical thickness at the hip (r = 0.410, *p* < 0.001) (Table 4).

## 4. Discussion

In this study on the bone properties of patients with IBD, their TBS scores did not show a significant difference compared to those of healthy controls matched by gender, age, and BMI. The analysis of TBS in adult patients with IBD has been previously evaluated in two cohort studies with discordant results. Krajcovicova et al. [19] included 50 patients with CD and 25 healthy controls matched for age, sex, weight, and vitamin D status. Consisting with our findings, no significant differences were found in mean TBS values between the CD and control cohorts (1.370 vs. 1.380, respectively). However, they observed a lower TBS value in the more severe CD group (Montreal classification B2 or B3, or treated with anti-TNFα), compared with the less severe CD patients (B1). Similar to our findings, areal BMD at the lumbar spine did not differ between the two cohorts or within the group of CD patients with different severity presentations [19]. In a more recent cohort study that enrolled 81 IBD adult patients and 81 controls, Soare et al. [20] observed statistically significant lower values of TBS in IBD patients compared to controls (1.380 vs. 1.430, respectively), as well as in the patients with stricturing CD compared with those with non-stricturing CD [20]. In addition to having a larger number of patients, the IBD cohort in Soare’s study included a higher proportion of CD subjects (59% of the sample) with more severe and active disease, as well as subjects with more frequent glucocorticoid exposure [20]. These factors may explain the different findings compared to those of our study. Finally, in a retrospective study of 50 children and adolescents with IBD aged 13.8 ± 3.0 years, the mean TBS in patients with CD was lower than in patients with UC (1.340 vs. 1.395), with no differences in terms of areal BMD values [34].

In a recent meta-analysis including 1338 IBD patients and 808 controls, it was reported that there was a significant decrease in areal BMD in IBD patients at the proximal femur and the lumbar spine [2]. Two more recent studies also demonstrate lower aBMD in patients with IBD compared to controls [20,35]. However, in agreement with previous studies [19,34], our analysis of aBMD using DXA at several anatomical sites, including lateral spine, did not show any significant differences between IBD patients and the control group. Additionally, we analyzed volumetric BMD using 3D-DXA, an emerging technique that provides information on bone structural properties and volumetric density of the cortical and the trabecular bone at the hip level. To the best of our knowledge, there are no published data on this technique in patients with IBD. As with areal DXA, we did not find any statistically significant difference in the results of the total trabecular, cortical, and integral volumetric BMD nor those of the cortical thickness in patients with IBD compared with healthy controls.

Regarding the lack of differences in our BMD-related results, it is worth noting that in our series, 70% of the patients with CD presented a B1 disease pattern (non-penetrating, non-stricturing), with only two cases (10%) presenting stricturing (B2), and four cases (19%) presenting penetrating CD disease (B3). Moreover, 72% of the patients with UC were in remission, and only one patient had severe colitis (S3). In terms of drug treatment, only 22% of IBD patients were receiving a biologic drug, and the current use of glucocorticoids was limited. Overall, these results reflect that our IBD population included mostly patients with mild or quiescent disease. Moreover, there was a significant use of vitamin D supplements, along with an adequate intake of dietary calcium in the IBD group. All these factors may explain why their TBS, aBMD, and vBMD and the cortical thickness at the hip were not negatively affected enough to find significant differences, in contrast to previous series where more severe patients were enrolled [2,20].

In contrast to DXA-derived assessments, the evaluation of QUS resulted in a significant difference between both groups, with BUA being lower in IBD patients. Moreover, the analysis, by splitting the two disease categories in the IBD group, yielded statistically significant lower values of BUA and BQI in the CD group compared with the healthy control group. Our results confirm the findings of several studies that have analyzed the utility of QUS in IBD in children and adults, suggesting that QUS may provide information on bone quality in addition to DXA and likely reflects the effect of the disease on bone microarchitecture [36,37,38]. The calcaneal QUS variables are related to bone density, trabecular orientation, the proportion of trabecular and cortical bone, the composition of organic and inorganic components, and fatigue damage of the bone [39,40]. As such, they reflect bone microarchitectural and composition characteristics that cannot be measured by DXA. TBS also provides information about the microarchitecture of the trabecular bone in the spine. This information results from various factors such as the disposition of the trabeculae, the arrangement of the lamellae, and collagen organization, which may be affected differently compared to QUS [40,41], in addition to the fact that the measurement is made at a different skeletal site.

In our study, we found a positive correlation between QUS values and all the other bone parameters derived from areal DXA and 3D-DXA. The strongest positive correlations were observed between the QUS parameters (BUA, SOS, and BQI) and the lumbar -lateral and anterior-posterior, femoral, and total hip areal BMD values (r = 0.27 to 0.59) as well as with the 3D-DXA parameters (cortical thickness, trabecular, cortical, and integral total vBMD) (r = 0.29 to 0.56). These results agree with those of previous reports that showed a positive correlation between QUS and DXA (r = 0.35–0.67) [36,37,42,43,44]. Interestingly, in contrast with QUS parameters, TBS values were not correlated with these DXA-derived parameters in our series. Finally, although a weak positive statistically significant correlation between BUA and TBS was observed for the full cohort of patients (r = 0.205, *p* = 0.047), this association was not confirmed after excluding one outlier per the predefined statistical analysis plan (r = 0.133, *p* = 0.205). Nevertheless, previous studies in postmenopausal women have reported significant correlations between QUS and TBS, with r = 0.177 [41] and r = 0.472 [40]

The estimation of the 10-year fracture risks with the FRAX tool adding the areal BMD data did not yield any increased risks in patients with IBD versus healthy controls, and these results remained non-significant when TBS was included in the risk assessment tool. However, when we segregated patients with IBD into CD and UC, we observed a statistically significant increased risk in those patients with CD for hip fracture (median: 0.8% in CD vs. 0.3% in UC and 0.4% in healthy controls (*p* = 0.045). For major osteoporotic fractures (MOFs), the 10-year risk results were at the limit of statistical significance (median: 3.1% in CD, 1.8% in UC and 2.2% for healthy controls (*p* = 0.050). These calculated fracture risk probabilities are lower than those described in other studies in patients with IBD, probably reflecting the relatively mild severity of the IBD population we enrolled in our study, as previously discussed. Thus, a systematic review that analyzed 1436 patients with IBD reported a 10-year risk for hip fracture of 1.03% and for MOFs of 4.05% [45]. These percentages were higher when only patients with CD were considered: 1.74% and 6.65% for hip and MOFs, respectively [45]. The risk factors included in the FRAX tool and analyzed in our study did not differ between patients with IBD and controls, except for the use of corticosteroids, which was absent in the control group, as it was an exclusion criterion, and was present in 12% of the IBD group.

No significant differences were observed in bone turnover marker levels between patients with IBD and healthy controls. Current evidence on bone turnover markers in the context of inflammatory bowel disease is limited, and available data on their diagnostic or monitoring utility remain controversial. While some studies have evaluated the response of bone turnover markers to treatments such as glucocorticoids or intravenous iron in IBD patients [46,47], the baseline status of markers such as P1NP or β-CTX compared to healthy controls has not been clearly established. We found a lower albumin level in the group of IBD; however, although this difference was statistically significant, it is not clinically relevant and is probably justified by the relationship between albumin levels and the state of intestinal inflammation. Unfortunately, we do not have calprotectin data collected to be able to support this hypothesis.

One strength of this study is its single-center assessment, which allowed for lower variability in bone assessment techniques and greater consistency in diagnosis, treatments, and procedures. Despite the relatively small sample size, the recruitment of a gender-, age-, and BMI-matched control group enabled the formation of two very homogeneous groups. Our study is the first to report lateral spine DXA and 3D-DXA outcomes using an approach based on 3D QCT-like models of the proximal femur and BMD distribution in patients with IBD. As limitations, the analyzed IBD patients presented with a low level of disease activity and severity, which may limit the findings of bone involvement. In particular, the very small number of patients with prevalent fractures did not allow us to evaluate whether TBS and the other bone assessments could be good predictive parameters. Another limitation of this study is that the power calculation was performed considering TBS. Therefore, the results of all the other secondary parameters investigated should be interpreted with caution. The lack of assessment of proinflammatory cytokines did not allow to correlate their relationship with the bone quality parameters. Finally, the number of cases of CU and CD are relatively small; thus, the study was underpowered for a formal comparison of the secondarily analyzed bone quality parameters between these two entities.

In conclusion, we have not observed a clinically significant lower bone quality in our cohort of IBD patients versus healthy controls using TBS, 3D-DXA, and aBMD, probably due to the low disease severity in the included patients. However, QUS parameters seem to be affected, especially in the CD subgroup. QUS has been shown to be a simple, low-cost, radiation-free, and safe test with a fracture prediction ability similar to that of DXA [16,18], and with significant correlations with other bone assessment tests, including 3D-DXA and aBMD. Hence, we feel that calcaneal QUS may be a valid screening test for metabolic bone disease in IBD patients.

## Figures and Tables

**Table 1:** Demographic characteristics, risks factors, laboratory findings, and FRAX.

Variables	IBD (n = 50)	UC (n = 29)	CD (n = 21)	Controls (n = 50)	*p* Value (IBD vs. Controls)	*p* Value Controls vs. UC vs. CD
**Demographic Characteristics**						
Sex (male/female)	20/30	12/17	8/13	20/30	1.000	0.973
Age (years)	52.0 ± 13.3	50.4 ± 11.9	54.3 ± 15.1	52.0 ± 14.2	0.988	0.608
BMI (kg/m^2^)	25.4 ± 4.1	25.6 ± 4.4	25.0 ± 3.8	26.1 ± 4.3	0.387	0.604
Weight (kg)	72.7 ± 16.4	74.2 ± 17.1	70.7 ± 15.4	74.0 ± 15.8	0.687	0.693
Height (cm)	168.6 ± 10.5	169.4 ± 9.9	167.5 ± 11.5	167.9 ± 9.5	0.721	0.756
**Risks Factors**						
Currently smokers	9 (18)	5 (17)	4 (19)	8 (16)	0.790	0.099
Alcohol ≥3 units/day	0 (0)	0 (0)	0(0)	3 (6)	0.242	1.000
Menopause	13 (43)	7 (41)	6 (46)	13 (43)	1.000	0.964
Previous fracture	3 (6)	1 (3)	2 (10)	0 (0)	0.242	0.098
Hip fracture history in parents	3 (6)	2 (7)	1 (5)	5 (10)	0.715	1.000
Use of calcium supplement	5 (10)	2 (7)	3 (14)	3 (6)	0.715	1.000
Use of vitamin D supplement	19 (38)	9 (31)	10 (48)	21 (42)	0.683	0.458
Calcium intake (mg/day)	837.9 ± 357.1	762.6 ± 223.2	941.8 ± 472.4	854.2 ± 277.8	0.799	0.141
**Laboratory findings**						
Albumin (g/dL)	4.60 (4.40; 4.70)	4.60 (4.40; 4.70)	4.60 (4.50; 4.70)	4.80 (4.60; 4.90)	< 0.001	0.003
Albumin corrected calcium (mg/dL)	9.19 ± 0.40	9.23 ± 0.32	9.14 ± 0.50	9.00 ± 0.33	0.012	0.029
Phosphorus (mg/dL)	3.6 ± 0.5	3.6 ± 0.5	3.4 ± 0.5	3.4 ± 0.4	0.220	0.121
Magnesium (mg/dL)	2.0 (1.9; 2.2)	2.0 (1.9; 2.1)	2.1 (1.9; 2.2)	2.1 (2.0; 2.2)	0.273	0.230
GFR (mL/min/1.73 m^2^)	94.5 ± 17.9	97.3 ± 14.2	90.7 ± 21.9	91.1 ± 16.0	0.313	0.234
iPTH (pg/mL)	52.8 ± 20.7	49.1 ± 19.6	57.8 ± 21.5	52.2 ± 15.1	0.887	0.219
ß-CTX (ng/mL)	0.340 (0.219; 0.444)	0.340 (0.230; 0.420)	0.350 (0.210; 0.440)	0.335 (0.250; 0.460)	0.634	0.870
P1NP (ng/mL)	43.7 (35.8; 69.5)	43.6 (37.3; 70.6)	44.4 (30.8; 57.3)	36.9 (33.5; 54.3)	0.104	0.207
25-OH-VitaminD (ng/mL)	24.0 (21.0; 32.0)	24.5 (21.0; 33.5)	24.0 (20.0; 32.0)	29.0 (20.0; 37.0)	0.314	0.518
Alkaline phosphatase (UI/L)	63.5 (53.0; 83.0)	59.0 (54.0; 82.0)	67.0 (48.0; 83.0)	64.0 (55.0; 73.0)	0.809	0.972
Urinary calcium/creatinine (mg/g)	0.11 (0.05; 0.20)	0.13 (0.08; 0.24)	0.07 (0.05; 0.16)	0.14 (0.10; 0.21)	0.378	0.218
FRAX *	**IBD (n = 41)**	**UC (n = 24)**	**CD (n = 17)**	**Controls (n = 40)**		
Hip fracture (%)	0.8 (0.1; 1.1)	0.3 (0.1; 0.6)	0.8 (0.4; 1.3)	0.4 (0.1; 0.7)	0.328	**0.045**
MOF (%)	2.2 (1.6; 4.2)	1.8 (1.4; 3.1)	3.1 (2.2; 4.3)	2.2 (1.4; 3.5)	0.499	**0.050**
TBS Hip Fracture (%)	0.3 (0.1; 1.1)	0.3 (0.1; 0.5)	0.9 (0.2; 1.8)	0.4 (0.1; 0.8)	0.559	0.066
TBS MOF (%)	2.7 (1.7; 4.7)	2.4 (1.5; 4.0)	4.2 (2.1; 5.7)	2.6 (1.6; 4.3)	0.441	0.128

Values presented are numbers (%) for categorical variables and mean ± SD or median (IQR) for continuous variables. “*p* value” was from Fisher’s exact test and Chi-square test for categorical variables and Student’s *t*-test, Mann–Whitney u-test, or ANOVA for continuous variables. * FRAX calculated only for ≥ 40 years old subjects. TBS Hip Fracture/MOF represents hip fracture and major osteoporotic fracture FRAX adjusted for TBS values, respectively. Abbreviations: IBD (inflammatory bowel disease), UC (ulcerative colitis), CD (Crohn’s disease), BMI (body mass index), GFR (glomerular filtration rate), iPTH (intact parathyroid hormone), ß-CTX (ß-isomer of carboxy-terminal telopeptide of type I collagen), P1NP (N-terminal propeptide of type I procollagen), MOF (major osteoporotic fracture), SD (standard deviation), and IQR (interquartile range).

**Table 2:** Clinical characteristics of patients with IBD.

Variables	IBD (n = 50)	UC (n = 29)	CD (n = 21)
Disease duration (years)	10.6 ± 11.7	11.3 ± 11.3	9.6 (12.4)
Therapies at baseline n (%)			
Mesalamine	29 (58)	23 (79)	6 (29)
Biologic therapies	11 (22)	3 (10)	8 (38)
Thiopurines	8 (16)	2 (7)	5 (24)
Non-systemic glucocorticoids	10 (20)	4 (14)	6 (29)
Systemic glucocorticoids	6 (12)	2 (7)	4 (19)
Prior surgery n (%)	4 (8)	0 (0)	4 (19)
Non-systemic corticosteroid (past users) n (%)	10 (20)	4 (14)	6 (29)
Systemic corticosteroid (past users) n (%)	27 (54)	15 (52)	12 (57)
Cumulative prednisone-equivalent dose (g)	3.28 ± 2.09	3.46 ± 2.55	3.07 ± 1.41
UC Montreal classification n (%)			
E1		11 (38)	
E2		9 (31)	
E3		9 (31)	
S0		21 (72)	
S1		3 (10)	
S2		4 (14)	
S3		1 (3)	
CD Montreal classification n (%)			
A1			1 (5)
A2			7 (33)
A3			13 (62)
L1			13 (62)
L2			2 (10)
L3			6 (29)
L4			0 (0)
B1			15 (71)
B2			2 (10)
B3			4 (19)

Values presented are numbers (%) for categorical variables and mean ± SD for continuous variables. Biologic therapies included anti-TNFα, anti-IL12-23, and anti-integrin. Non-systemic glucocorticoids included topic, budesonide, and fluticasone dipropionate. Abbreviations: IBD (inflammatory bowel disease), UC (ulcerative colitis), and CD (Crohn’s disease). The IBD Montreal classification criteria are included in Supplementary Tables S1 and S2 [1].

**Table 3:** Comparison of bone assessment techniques between IBD and healthy control group.

Variables	IBD (n = 50)	UC (n = 29)	CD (n = 21)	Controls (n = 50)	*p* Value (IBD vs. controls)	*p* Value Controls vs. UC vs. CD
**TBS** (unitless)	1.296 ± 0.120	1.305 ± 0.116	1.283 ± 0.126	1.318 ± 0.103	0.322	0.481
**aBMD (DXA)**						
L2-L4 lat (g/cm^2^)	0.720 ± 0.237	0.741 ± 0.263	0.691 ± 0.194	0.776 ± 0.242	0.254	0.409
L1-L4 (g/cm^2^)	1.120 ± 0.160	1.135 ± 0.174	1.100 ± 0.141	1.138 ± 0.171	0.603	0.633
Femoral Neck (g/cm^2^)	0.898 ± 0.149	0.910 ± 0.136	0.881 ± 0.167	0.922 ± 0. 136	0.409	0.557
Total Hip (g/cm^2^)	0.941 ± 0.151	0.964 ± 0.131	0.908 ± 0.173	0.972 ± 0.145	0.296	0.246
**vBMD (3D-DXA)**						
Integral total (g/cm^3^)	313.17 (283.64; 335.01)	318.60 (294.97; 327.04)	296.82 (270.97; 335.01)	313.79 (284.61; 360.47)	0.397	0.484
Cortical total (g/cm^3^)	818.07 ± 71.33	829.17 ± 62.74	802.73 ± 80.81	827.23 ± 80.26	0.549	0.400
Trabecular total (g/cm^3^)	162.78 ± 38.98	165.80 ± 35.09	158.60 ± 44.34	173.53 ± 43.24	0.197	0.363
Cortical thickness (mm)	1.926 ± 0.177	1.938 ± 0.148	1.910 ± 0.214	1.948 ± 0.153	0.520	0.686
**QUS**						
BUA (db/Mhz)	107.2 (93.3; 114.3)	108.6 (99.0; 118.2)	96.7 (90.1; 110.9)	108.9 (100.8; 123.0)	0.083	**0.030**
BUA (db/Mhz) w/o outliers *	103.6 ± 14.3	106.8 ± 14.7	99.5 ± 12.8	111.3 ± 19.5	**0.033**	**0.030**
SOS (m/s)	1512.9 (1501.9; 1522.5)	1519.1 (1502.6; 1525.9)	1509.7 (1497.9; 1514.3)	1513.5 (1501.6; 1527.5)	0.501	0.142
BQI (%)	86.9 ± 14.9	91.4 ± 14.3	80.7 ± 13.7	92.2 ± 20.3	0.151	**0.041**
T-Score and Z-Score DXA **						
T-score L1-L4	−0.93 ± 1.30	−0.92 ± 1.47	−0.93 ± 1.10	−0.68 ± 1.47	0.475	0.777
Z-score L1-L4	−0.07 ± 1.24	0.08 ± 1.34	−0.35 ± 1.07	−0.25 ± 1.23	0.659	0.722
T-score Femoral Neck	−1.50 (−2.00; −0.20)	−1.50 (−2.00; −0.50)	−1.50 (−2.00; −0.10)	−1.1 (−1.70; −0.70)	0.423	0.835
Z-score Femoral Neck	−0.38 ± 0.96	−0.13 ± 0.98	−0.88 ± 0.77	0.01 ± 1.29	0.321	0.254
T-score Total Hip	−0.98 ± 1.21	−0.89 ± 1.11	−1.07 ± 1.34	−0.78 ± 1.04	0.471	0.699
Z-score Total Hip	−0.58 ± 0.87	−0.38 ± 0.76	0.97 ± 1.03	−0.05 ± 1.18	0.145	0.187
**Osteoporosis n (%)**	10 (20)	5 (17)	5 (24)	3 (6)	**0.037**	0.091

Values presented are mean ± SD or median (IQR) as appropriate. “*p* value” was from Student’s *t*-test or Mann–Whitney U test. vBMD: controls n = 49 for one missing value. QUS: UC n = 27 and CD n = 20 for three missing values; and controls n = 47 for three missing values. * One outlier (UC group) with BUA value 178.6 db/Mhz (+3.73 SD). ** T-score was calculated for patients aged 50 years or more and Z-score for patients aged below 50 years. Controls ≥ 50 years n = 33, IBD ≥ 50 years n = 32. Controls < 50 years n = 16, IBD < 50 years n = 18. Abbreviations: aDMO (areal bone mineral density), DXA (dual energy X-ray absorptiometry), vBMD (volumetric bone mineral density), QUS (quantitative bone ultrasound), BUA (broadband ultrasound attenuation), w/o (without), SOS (speed Of sound), BQI (bone quality index), and y/o (years old).

**Table 4:** Correlations between bone assessment techniques.

Variables	Pearson Correlation (r)	*p* Value	Pearson Correlation (r) (w/o Outliers)	*p* Value (w/o Outliers)
TBS vs. SOS	0.073	0.480		
TBS vs. BUA	**0.205 ^a^**	**0.047**	0.133	0.205
TBS vs. BQI	0.118	0.254		
TBS vs. L1-L4 aBMD	0.118	0.240		
TBS vs. L2-L4 lat aBMD	−0.006	0.955		
TBS vs. Femoral Neck aBMD	0.174	0.083		
TBS vs. Total Hip aBMD	0.060	0.553		
TBS vs. vBMD integral	0.069	0.497		
TBS vs. vBMD cortical	0.063	0.537		
TBS vs. vBMD trabecular	0.146	0.148		
TBS vs. Cortical Thickness	−0.097	0.338		
SOS vs. vBMD integral	**0.528**	**<0.001**		
SOS vs. vBMD cortical	**0.507**	**<0.001**		
SOS vs. vBMD trabecular	**0.558**	**<0.001**		
SOS vs. Cortical Thickness	**0.474**	**<0.001**		
BUA vs. vBMD integral	**0.293**	**0.004**	**0.314 ^a^**	**0.002**
BUA vs. vBMD cortical	0.192	0.065	**0.286**	**0.006**
BUA vs. vBMD trabecular	**0.271**	**0.009**	**0.305**	**0.003**
BUA vs. Cortical Thickness	**0.335**	**0.001**	**0.410**	**<0.001**
BQI vs. vBMD integral	**0.491**	**<0.001**		
BQI vs. vBMD cortical	**0.449**	**<0.001**		
BQI vs. vBMD trabecular	**0.524**	**<0.001**		
BQI vs. Cortical Thickness	**0.488**	**<0.001**		
SOS vs. L1-L4 aBMD	**0.506**	**<0.001**		
SOS vs. L2-L4 lat aBMD	**0.515**	**<0.001**		
SOS vs. Femoral Neck aBMD	**0.513**	**<0.001**		
SOS vs. Total Hip aBMD	**0.592**	**<0.001**		
BUA vs. L1-L4 aBMD	0.194	0.061	**0.268**	**0.009**
BUA vs. L2-L4 lat aBMD	**0.333**	**0.001**	**0.427**	**<0.001**
BUA vs. Femoral Neck aBMD	**0.343** **^a^**	**<0.001**	**0.365 ^a^**	**<0.001**
BUA vs. Total Hip aBMD	**0.326**	**0.001**	**0.403**	**<0.001**
BQI vs. L1-L4 aBMD	**0.450**	**<0.001**		
BQI vs. L2-L4 lat aBMD	**0.515**	**<0.001**		
BQI vs. Femoral Neck aBMD	**0.501**	**<0.001**		
BQI vs. Total Hipt aBMD	**0.569**	**<0.001**		

^a^ Rho de Spearman. Bold values denote significant values *p* < 0.05. Outliers were defined as those that were within ±3 SD of the mean. Abbreviations: w/o (without), TBS (trabecular bone score), SOS (speed of sound), BUA (broadband ultrasound attenuation), BQI (bone quality index), L1–L4 (lumbar spine L1–L4), L2–L4 lat (lateral lumbar spine L2–L4), aBMD (areal bone mineral density), and vBMD (volumetric bone mineral density).

## Data Availability

The data are not publicly available due to ethical restrictions and the need to protect patient confidentiality.

## Supplementary Materials

The following supporting information can be downloaded at: https://www.mdpi.com/article/10.3390/jcm14113933/s1, Table S1. Montreal classification of Crohn’s disease. Table S2. Montreal classification of Ulcerative Colitis.

## Abbreviations

The following abbreviations are used in this manuscript:

IBD	inflammatory bowel disease
DXA	dual-energy X-ray absorptiometry
aBMD	areal bone mineral density
VFA	vertebral fracture assessment
TBS	trabecular bone score
QUS	calcaneal quantitative ultrasound
vBMD	volumetric bone mineral density
UC	ulcerative colitis
CD	Crohn’s disease
BUA	broadband ultrasound attenuation
TNF-α	tumor necrosis factor alpha
IL	interleukin
BMD	bone mineral density
BMI	body mass index
HF	hip fracture
MOF	major osteoporotic fractures
CV	coefficient of variation
vBMD	volumetric bone mineral density
LSC	least significant changes
SOS	speed of sound
BQI	bone quality index
iPTH	intact parathyroid hormone
ß-CTx	ß-isomer of carboxy-terminal telopeptide of type I collagen
P1NP	N-terminal propeptide of type I procollagen
SD	standard deviation
IQR	interquartile range

## References

[1] Silverberg M.S., Satsangi J., Ahmad T., Arnott I.D., Bernstein C.N., Brant S.R., Caprilli R., Colombel J.F., Gasche C., Geboes K. (2005). Toward an integrated clinical, molecular and serological classification of inflammatory bowel disease: Report of a working party of the 2005 Montreal world congress of gastroenterology. Can. J. Gastroenterol..

[2] Szafors P., Che H., Barnetche T., Morel J., Gaujoux-Viala C., Combe B., Lukas C. (2018). Risk of fracture and low bone mineral density in adults with inflammatory bowel diseases. A systematic literature review with meta-analysis. Osteoporos. Int..

[3] Komaki Y., Komaki F., Micic D., Ido A., Sakuraba A. (2019). Risk of fractures in inflammatory bowel diseases: A systematic review and meta-Analysis. J. Clin. Gastroenterol..

[4] Kärnsund S., Lo B., Bendtsen F., Holm J., Burisch J. (2020). Systematic review of the prevalence and development of osteoporosis or low bone mineral density and its risk factors in patients with inflammatory bowel disease. World J. Gastroenterol..

[5] Lo B., Holm J.P., Vester-Andersen M.K., Bendtsen F., Vind I., Burisch J. (2020). Incidence, risk factors and evaluation of osteoporosis in patients with inflammatory bowel disease: A Danish population-based inception cohort with 10 years of follow-up. J. Crohns Colitis..

[6] Van Bodegraven A.A., Bravenboer N. (2020). Perspective on skeletal health in inflammatory bowel disease. Osteoporos. Int..

[7] Targownik L.E., Bernstein C.N., Leslie W.D. (2014). Risk factors and management of osteoporosis in inflammatory bowel disease. Curr. Opin. Gastroenterol..

[8] Briot K., Geusens P., Em Bultink I., Lems W.F., Roux C. (2017). Inflammatory diseases and bone fragility. Osteoporos. Int..

[9] Vestergaard P., Mosekilde L. (2002). Fracture risk in patients with celiac disease, Crohn’s disease, and ulcerative colitis: A nationwide follow-up study of 16,416 patients in Denmark. Am. J. Epidemiol..

[10] Ewid M., Mutiri N.A., Omar K.A., Shamsan A.N., Rathore A.A., Saquib N., Salaas A., Al Sarraj O., Nasri Y., Attal A. (2020). Updated bone mineral density status in Saudi patients with inflammatory bowel disease. World J. Gastroenterol..

[11] Jahnsen J., Falch J.A., Aadland E., Mowinckel P. (1997). Bone mineral density is reduced in patients with Crohn’s disease but not in patients with ulcerative colitis: A population based study. Gut.

[12] Rosen H.N., Vokes T.J., Malabanan A.O., Deal C.L., Alele J.D., Olenginski T.P., Schousboe J.T. (2013). The official positions of the international society for clinical densitometry: Vertebral fracture assessment. J. Clin. Densitom..

[13] Beck T.J., Broy S.B. (2015). Measurement of hip geometry—Technical background. J. Clin. Densitom..

[14] Humbert L., Martelli Y., Fonolla R., Steghofer M., Di Gregorio S., Malouf J., Romera J., Barquero L.M. (2017). 3D-DXA: Assessing the femoral shape, the trabecular macrostructure and the cortex in 3D from DXA images. IEEE Trans. Med. Imaging..

[15] Silva B.C., Leslie W.D., Resch H., Lamy O., Lesnyak O., Binkley N., McCloskey E.V., Kanis J.A., Bilezikian J.P. (2014). Trabecular bone score: A noninvasive analytical method based upon the DXA image. J. Bone Miner. Res..

[16] Marín F., González-Macías J., Díez-Pérez A., Palma S., Delgado-Rodríguez M. (2006). Relationship between bone quantitative ultrasound and fractures: A meta-analysis. J. Bone Miner. Res..

[17] Hans D., Goertzen A.L., Krieg M.A., Leslie W.D. (2011). Bone microarchitecture assessed by TBS predicts osteoporotic fractures independent of bone density: The Manitoba study. J. Bone Miner. Res..

[18] McCloskey E.V., Kanis J.A., Odén A., Harvey N.C., Bauer D., González-Macias J., Hans D., Kaptoge S., Krieg M.A., Kwok T. (2015). Predictive ability of heel quantitative ultrasound for incident fractures: An individual-level meta-analysis. Osteoporos. Int..

[19] Krajcovicova A., Kuzma M., Hlavaty T., Hans D., Koller T., Jackuliak P., Leskova Z., Sturdik I., Killinger Z., Payer J. (2018). Decrease of trabecular bone score reflects severity of Crohn’s disease: Results of a case–control study. Eur. J. Gastroenterol. Hepatol..

[20] Soare I., Sirbu A., Martin S., Diculescu M., Mateescu B., Tieranu C., Fica S. (2021). Assessment of bone quality with trabecular bone score in patients with inflammatory bowel disease. Sci. Rep..

[21] Humbert L., Winzenrieth R., Di Gregorio S., Thomas T., Vico L., Malouf J., Del Río Barquero L.M. (2019). 3D analysis of cortical and trabecular bone from hip DXA: Precision and trend assessment interval in postmenopausal women. J. Clin. Densitom..

[22] Hong A.R., Kim J.H., Kim S.W., Kim S.Y., Shin C.S. (2016). Trabecular bone score as a skeletal fragility index in acromegaly patients. Osteoporos. Int..

[23] Choi Y.J., Lee H.Y., Yoon D., Kim A., Shin Y.S., Park H.S., Ye Y.M. (2019). Trabecular bone score is more sensitive to asthma severity and glucocorticoid treatment than bone mineral density in asthmatics. Allergy Asthma Immunol. Res..

[24] Kužma M., Vaňuga P., Binkley N., Ságová I., Pávai D., Blažíček P., Kužmová Z., Jackuliak P., Vaňuga A., Killinger Z. (2018). High serum fractalkine is associated with lower trabecular bone score in premenopausal women with Graves’ disease. Horm. Metab. Res..

[25] Catalano A., Gaudio A., Agostino R.M., Morabito N., Bellone F., Lasco A. (2019). Trabecular bone score and quantitative ultrasound measurements in the assessment of bone health in breast cancer survivors assuming aromatase inhibitors. J. Endocrinol. Investig..

[26] Marques J.V.O., Nalevaiko J.Z., Oliveira M.F., Raetsch A.W.P., Marques G.L., Petterle R.R., Moreira C.A., Borba V.Z.C. (2020). Trabecular bone score (TBS) and bone mineral density in patients with long-term therapy with warfarin. Arch. Osteoporos..

[27] Kim Y.J., Kang K.Y., Shin J., Jun Y., Kim S.I., Kim Y.R. (2020). Trabecular bone scores in young HIV-infected men: A matched case-control study. BMC Musculoskelet. Disord..

[28] Stachowska B., Halupczok-Żyła J., Kuliczkowska-Płaksej J., Syrycka J., Bolanowski M. (2021). Decreased trabecular bone score in patients with active endogenous Cushing’s syndrome. Front. Endocrinol..

[29] Banaszkiewicz K., Sikorska K., Panas D., Sworczak K. (2021). The role of the trabecular bone score in the assessment of osteoarticular disorders in patients with HFE-hemochromatosis: A single-center study from Poland. Genes.

[30] Yokomoto-Umakoshi M., Umakoshi H., Sakamoto R., Fukumoto T., Ogata M., Nakano Y., Iwahashi N., Kaneko H., Mizoguchi N., Hattori A. (2021). Role of deteriorated bone quality in the development of osteoporosis in pheochromocytoma and paraganglioma. Bone.

[31] Olmos-Martínez J.M., Hernández J.L., Fábrega E., Olmos J.M., Crespo J., González-Macías J. (2020). Bone mineral density and trabecular bone score in treatment-naïve patients with non-cirrhotic hepatitis C virus infection. Arch. Osteoporos..

[32] Calatayud M., Pérez-Olivares Martín L., Librizzi S., Lora Pablos D., González Méndez V., Aramendi Ramos M., Martínez Diaz-Guerra G., Hawkins F. (2021). Trabecular bone score and bone mineral density in patients with long-term controlled acromegaly. Clin. Endocrinol..

[33] Kim E.H., Jeon Y.K., Pak K., Kang T., Kim K.E., Kim S.J., Kim I.J., Kim K. (2021). Effect of tamoxifen with or without gonadotropin-releasing hormone analog on DXA values in women with breast cancer. Sci. Rep..

[34] Levy-Shraga Y., Megnazi O., Modan-Moses D., Tripto-Shkolnik L., Gruber N., Haberman Y., Shouval D.S., Weiss B. (2021). Trabecular bone score in children and adolescents with inflammatory bowel diseases. J. Clin. Densitom..

[35] Pepe J., Zawadynski S., Herrmann F.R., Juillerat P., Michetti P., Ferrari-Lacraz S., Belli D., Ratib O., Rizzoli R., Chevalley T. (2017). Structural basis of bone fragility in young subjects with inflammatory bowel disease: A high-resolution pQCT study of the SWISS IBD Cohort (SIBDC). Inflamm. Bowel Dis..

[36] Robinson R.J., Carr I., Lqbal S.J., AI-Azzawi F., Abrams K., Mayberry J.F. (1998). Screening for osteoporosis in Crohn’s disease. A detailed evaluation of calcaneal ultrasound. Eur. J. Gastroenterol. Hepatol..

[37] Fries W., Dinca M., Luisetto G., Peccolo F., Bottega F., Martin A. (1998). Calcaneal ultrasound bone densitometry in inflammatory bowel disease--a comparison with double X-ray densitometry of the lumbar spine. Am. J. Gastroenterol..

[38] Turk N., Kastelan D., Cukovic-Cavka S., Kraljevic I., Korsic M., Vucelic B. (2007). Discriminatory ability of calcaneal quantitative ultrasound in the assessment of bone status in patients with inflammatory bowel disease. Ultrasound Med. Biol..

[39] Wear K.A. (2020). Mechanisms of interaction of ultrasound with cancellous bone: A review. IEEE Trans. Ultrason. Ferroelectr. Freq. Control.

[40] Métrailler A., Hans D., Lamy O., Gonzalez Rodriguez E., Shevroja E. (2023). Heel quantitative ultrasound (QUS) predicts incident fractures independently of trabecular bone score (TBS), bone mineral density (BMD), and FRAX: The OsteoLaus Study. Osteoporos. Int..

[41] Olmos J.M., Hernández J.L., Pariente E., Martínez J., Valero C., González-Macías J. (2020). Trabecular bone score and bone quantitative ultrasound in Spanish postmenopausal women. The Camargo Cohort Study. Maturitas.

[42] Jahnsen J., Falch J.A., Mowinck P. (1999). Ultrasound measurements of calcaneus for estimation of skeletal status in patients with inflammatory bowel disease. Scand. J. Gastroenterol..

[43] Heijckmann A.C., Dumitrescu B., Nieuwenhuijzen Kruseman A.C., Geusens P., Wolffenbuttel B.H., De Vries J., Drent M., Huijberts M.S. (2008). Quantitative ultrasound does not identify patients with an inflammatory disease at risk of vertebral deformities. BMC Musculoskelet. Disord..

[44] Schwartz D.A., Connolley C.D., Koyama T., Wise P.E., Herline A.J. (2005). Calcaneal ultrasound bone densitometry is not a useful tool to screen patients with inflammatory bowel disease at high risk for metabolic bone disease. Inflamm. Bowel Dis..

[45] Serrano-Montalbán B., Arias Á., Friginal-Ruiz A.B., Lucendo A.J. (2017). The use of the fracture risk assessment (FRAX^®^) tool in predicting risk of fractures in patients with inflammatory bowel disease: A systematic review. J. Clin. Densitom..

[46] Vihinen M.K., Kolho K.L., Ashorn M., Verkasalo M., Raivio T. (2008). Bone turnover and metabolism in paediatric patients with inflammatory bowel disease treated with systemic glucocorticoids. Eur. J. Endocrinol..

[47] Tulewicz-Marti E., Szwarc P., Więcek M., Lewandowski K., Korcz T., Cicha M., Rydzewska G. (2023). Effect of intravenous iron administration on bone mineral and iron homeostasis in patients with inflammatory bowel disease—Results of a prospective single-centre study. J. Pers. Med..

