# Supplementary material for: Assessment of Bone Health in Adult Patients with Inflammatory Bowel Disease: A Single-Center Cohort Study
Source: J Clin Med. 2025 Jun 3;14(11):3933. doi: 10.3390/jcm14113933 (PMC12155898; doi:10.3390/jcm14113933)
Supplement: Supplementary file 1 [file jcm-14-03933-s001.zip › jcm-3653289-supplementary.pdf]

Supplementary Materials:

Supplementary Table S1. Montreal classification of Crohn's disease

**Age at diagnosis (A)**	
A1	16 years or younger
A2	17–40 years
A3	Over 40 years
**Location (L)**	
L1	Terminal ileum
L2	Colon
L3	Ileocolon
L4	Upper gastrointestinal
**Behaviour (B)**	
B1	Nonstricturing, nonpenetrating
B2	Stricturing
B3	Penetrating
p	Perianal disease modifier

Silverberg MS, Satsangi J, Ahmad T, Arnott ID, Bernstein CN, Brant SR, et al. Toward an Integrated Clinical, Molecular and Serological Classification of Inflammatory Bowel Disease: Report of a Working Party of the 2005 Montreal World Congress of Gastroenterology. Can J Gastroenterol. 2005;19(suppl a):5A-36A.

Supplementary Table S2. Montreal classification of Ulcerative Colitis

**Extent**		
E1	Ulcerative proctitis	Limited to the rectum
E2	Left-side UC	Limited to the portion of the colorectum distal to the splenic flexure
E3	Extensive UC (pancolitis)	Extends proximal to the splenic flexure
**Severity**		
S0	UC in clinical remission	No symptoms of UC
S1	Mild UC	Four or fewer bloody stools daily, lack of fever, pulse of less than 90 beats/min, hemoglobin of 105 g/L or greater and erythrocyte sedimentation rate of less than 30 mm/h.
S2	Moderate UC	The state between mild and severe
S3	Severe UC	At least six bloody stools daily, pulse of at least 90 beats/min, temperature of at least 37.5°C, hemoglobin of less than 105 g/L and ESR of at least 30 mm/h

Abbreviations: UC (ulcerative colitis)

Silverberg MS, Satsangi J, Ahmad T, Arnott ID, Bernstein CN, Brant SR, et al. Toward an Integrated Clinical, Molecular and Serological Classification of Inflammatory Bowel Disease: Report of a Working Party of the 2005 Montreal World Congress of Gastroenterology. Can J Gastroenterol. 2005;19(suppl a):5A-36A.
